# Sustained mesenchymal reprogramming of endothelial cells after completion of chemotherapy

**DOI:** 10.1186/s40959-025-00413-7

**Published:** 2025-12-29

**Authors:** Kass Sjostrom, Shixin Tao, Melissa S. Cobb, Shannon Landers, Amy Kwok, Ria Singh, Ralph V. Shohet, Nataliya Kibiryeva, Eugene A. Konorev

**Affiliations:** 1 College of Biosciences, Kansas City University, Kansas City, USA; 2 College of Osteopathic Medicine, Kansas City University, 1750 Independence Ave, Kansas City, MO 64106 USA; 3 John A. Burns School of Medicine, University of Hawaii, Honolulu, USA; 4 College of Integrated Health Science, University at Albany, Albany, USA

**Keywords:** Endothelium, TGF-β pathway, Anthracyclines

## Abstract

Cardiovascular disease is the prevailing cause of death among cancer survivors. Remarkable increase in cardiac disease burden in this group is likely due to the effects of cancer itself and, especially, cardiotoxic cancer treatments. Of the cardiotoxic cancer treatments, anthracyclines are notoriously known for causing vascular damage and severe cardiovascular complications. A defining feature of cardiac damage by doxorubicin (Dox), a prototypical anthracycline, is that it typically progresses after completion of chemotherapy. As the nature of delayed deterioration is not understood, we focused in this study on the events that occur upon completion of chemotherapy. We adopted the Dox treatment/washout model to examine its lasting effects on endothelial cells. Our ChIP sequencing, transcriptomic, reporter plasmid, and Smad2/3 phosphorylation experiments demonstrated enhanced activity of the canonical TGF-β and activin pathways during Dox washout. Another notable feature was sustained mesenchymal reprogramming with significant upregulation of transcripts characteristic of fibroblastic and smooth muscle lineages, both in endothelial cultures and cardiac microvascular endothelial cells in vivo. We utilized a selective ALK4/5/7 receptor kinase inhibitor, SB431542 (SB), to probe the role of the canonical TGF-β/activin pathways in endothelial-to-mesenchymal reprogramming by Dox. When present during Dox washout, SB blocked increased expression of both mesenchymal transcripts and protein markers, and prevented cytoskeletal changes and fibronectin production by the treated endothelial cells. Cytoskeletal rearrangements led to increased endothelial monolayer permeability that was abolished by SB treatment. Thus, increased production of ALK4/5 receptor ligands, TGF-β2 and activin, and heightened Smad2/3 activation response to these ligands during Dox washout leads to sustained mesenchymal reprogramming of endothelial cells and compromised endothelial barrier function.

**Supplementary Information:**

The online version contains supplementary material available at 10.1186/s40959-025-00413-7.

## Background

The number of cancer survivors has increased substantially over the past decades due to both population aging and advances in early detection and treatment [1, 2]. In 2022, 70% of cancer survivors have lived 5 years or more after diagnosis, and this cohort now accounts for around 5% of the US population [3, 4]. The success in the treatment and even cure of cancer is overshadowed by increased mortality of cancer survivors from non-cancer causes, which exceeded mortality of cancer related causes in this cohort [5]. In particular, these patients more often die of cardiovascular diseases than cancer [5, 6].

The remarkable increase in cardiac disease burden in this group is in part due to cardiotoxic cancer treatments [7, 8]. Of these treatments, anthracyclines are notoriously known for causing severe cardiovascular complications [9, 10]. Doxorubicin (Dox), a prototypical anthracycline, can cause cardiomyopathy in treated patients, with most cases occurring within one year after completion of the treatment [11]. Subclinical cardiovascular damage often persists after completion of therapy and may eventually progress to heart failure, especially in patients with cardiovascular comorbidities [7]. A defining feature of Dox cardiomyopathy is that it typically develops after the treatment. The nature of delayed deterioration is not understood. In order to understand the mechanisms of these delayed detrimental effects of Dox, it is important to focus on events that occur upon completion of chemotherapy.

Several recent studies have brought attention to the role of endothelial cells in cardiac diseases, including heart failure [12, 13]. Besides the obvious function of supplying oxygen, microvasculature in the heart produces angiocrine factors that maintain cardiomyocyte function and metabolism [14]. Microvascular endothelial cells develop from heterogeneous pools of progenitors [15] and produce unique sets of angiocrine factors [16]. Specific ablation of such protective endothelial derived factors is sufficient to cause heart failure [17, 18]. Experimental evidence is now emerging that endothelial dysfunction may contribute to Dox cardiomyopathy [19, 20]. In investigating this hypothesis, we observed increased nuclear accumulation of Dox in murine cardiac endothelial cells, as compared with non-endothelial cardiac cells, after intravenous administration of the drug [21]. These studies suggest that endothelial cells likely constitute a critical target of Dox in the heart. Indeed, Dox induced damage to both coronary arteries and cardiac microvessels has been described in both animals and treated cancer patients [21–23]. Importantly, damage to cardiac vasculature preceded contractile decline in Dox treated animals [24, 25] and progressively deteriorated after completion of therapy [21, 25]. Protective interventions that specifically targeted endothelial cells alleviated Dox cardiotoxicity [26] further implicating the role of the endothelium in developing cardiomyopathy.

Multiple mechanisms of endothelial cell damage by Dox have been described [19]. Our previous in vitro and in vivo studies have highlighted the role of the TGF-β pathway in endothelial damage by Dox [21, 27]. Increase in plasma TGF-β levels has been detected in patients after Dox, other chemotherapeutic drugs, and radiation treatments [28–30]. Furthermore, elevation in plasma TGF-β concentration in this setting strongly correlated with the risk of developing post-treatment complications [29, 31, 32]. We have previously described increased expression of the TGF-β family members in both cultured cardiac endothelial cells treated with Dox and hearts of Dox treated mice [21, 27]. In addition, using both pharmacological and gene knockout strategies, we have provided evidence that endothelial damage, cardiovascular remodeling and dysfunction by Dox are mediated by the activated TGF-β/Smad3 pathway [21, 27].

While we have demonstrated involvement of the TGF-β/Smad3 pathway in vascular damage by Dox, the downstream processes remain unknown. TGF-β has been implicated in endothelial-to-mesenchymal transition (EndMT) in several vascular pathologies, including atherosclerosis, transplant vasculopathy, and degeneration of renal microvasculature [33–36]. In addition to enhancing the TGF-β pathway, Dox was found in our previous studies [21] to suppress activity of vascular endothelial growth factor (VEGF) and hepatocyte growth factor (HGF), the pathways that prevent EndMT and maintain endothelial homeostasis [37–39]. Furthermore, inflammation and oxidative stress that promote EndMT are upregulated by Dox therapy [21, 35, 40]. In this study, we tested the hypothesis that Dox causes mesenchymal reprogramming in endothelial cells via the canonical TGF-β pathway. In the context of this study, we define mesenchymal reprogramming as activation of mesenchymal gene transcription in endothelial cells in the process of partial EndMT. In order to model the changes that persist after completion of chemotherapy, we examined endothelial transcriptomes, protein expression, and function after Dox was removed from the culture media.

## Methods

### Animals, cell lines, and reagents

Mice of the mixed B6;129 or C57BL6J backgrounds were purchased from the Jackson Laboratory (strains #100903 and #000664, respectively), housed and bred at the University of Missouri-Kansas City Laboratory Animal Research facility. Mice were fed a standard chow, Teklad Global 2918 diet, and assigned randomly to experimental groups. Animal experiments were performed in accordance with National Institutes of Health *Guide for the Care and Use of Laboratory Animals* and approved by the Kansas City University and the University of Missouri-Kansas City Institutional Animal Care and Use Committees. Dox cardiomyopathy was modelled in 8 to 11-week-old male and female C57BL/6 J mice using four injections of Dox (5 mg/kg) or vehicle (saline) via the tail vein over two consecutive weeks, for a total Dox dose of 20 mg/kg. Mice were sacrificed for immunohistochemistry analysis three weeks after the last injection of Dox. To evaluate accumulation of Dox in cardiac endothelial cells, 10 to 15-week-old male B6;129 mice were injected via the tail vein with 5 mg/kg doxorubicin, anesthetized with isoflurane (3% mixed with 1L/min O2 via a facemask) and then intravenously injected with the vital dye isolectin B4-DyLight 649 (Vector Laboratories, 100 µl of 1 mg/ml solution per injection) to label endothelial cells. Pooled human umbilical vein endothelial cells (HUVEC, C2519A) and human cardiac microvascular endothelial cells (HCMVEC, CC-7030) were purchased from Lonza and cultured in EGM2 and EGM2-MV complete media, respectively, that were prepared using endothelial basal media (EBM2, CC-3156) and SingleQuot supplements (CC-4176 or CC-4147). Cell lines were authenticated using double positivity for CD31/CD105 endothelial marker proteins and cobblestone cell morphology and tested negative for mycoplasma. We used the following primary antibodies against transgelin (Abcam, ab14106, for immunoblotting and immunochemistry); β-actin (ThermoFisher, AM4302, for immnunoblotting); phospho-Smad2/3 (Abcam, ab52903 for immunoblotting); Smad2 (Cell Signaling Technology, 5339, for immunoblotting); Smad3 (ThermoFisher, MA5-14,939, for immunoblotting, and #9523 for chromatin immunoprecipitation); fibronectin (Millipore-Sigma, F0916, for immunoblotting, and Proteintech, 15,613–1-AP, for immunochemistry); N-cadherin (ThermoFisher, MA1-2002, for immunoblotting); and CD31 (Dako, M0823, for immunocytochemistry). Secondary antibodies were as follows: goat anti-mouse Alexa Fluor 488 and goat anti-rabbit Alexa Flour 594 (both from ThermoFisher, A11017 and A11072, respectively, for immunocytochemistry); and IRDye 680LT goat anti-mouse and IRDye 800CW goat anti-rabbit (both from LI-COR, 926–68,020 and 926–32,211, respectively, for immunoblotting). Other key reagents were: Dox (Cell Signaling Technology, 5297); SB431542 (LC Laboratories, S-7800); TGF-β2 (R&D Systems, 302-B2); *Griffonia simplicifolia* isolectin B4-DyLight 649 (ILB4, Vector Laboratories, DL-1208); Texas Red X-conjugated phalloidin (ThermoFisher, T7471); and fluorophore conjugated dextrans (ThermoFisher, 10,000 MW Cascade Blue conjugated and 70,000 MW Texas Red conjugated, D1976 and D1864, respectively).

### Cell culture and treatments

All experiments with cultured endothelial cells were performed in passage 7. Cells were grown at 37°C and 5% CO_2_ and passaged using 0.05% Trypsin–EDTA that was subsequently neutralized with defined trypsin inhibitor (ThermoFisher Scientific). Smad3 deficient HCMVEC line was previously described [21]. Cultured endothelial cells were treated with 16 nM Dox, a sub-apoptotic concentration that was used in our previous studies to model exposure to the drug in treated patients and animals [21, 27]. Cells were used in downstream assays after Dox treatment (Dox treatment protocol) or after a period of the post-treatment culturing in Dox-free media (Dox washout protocol). A selective ALK4/5/7 receptor kinase inhibitor, SB431542 (1 µM) [41], was present during either Dox treatment only, or Dox washout only, or both. In separate experiments, control (scrambled shRNA) and Smad3 shRNA expressing cell lines were treated with 0.3 ng/ml TGF-β2 for 16 h before isolating total RNA for sequencing and qPCR.

### PCR and immunoblotting

To extract total RNA, cells were lysed with TRIzol and total RNA was isolated with the PureLink RNA kit (ThermoFisher Scientific). Complementary DNA was generated using a High Capacity cDNA synthesis kit (ThermoFisher Scientific) and quantitative PCR was performed using a CFX Connect Real-Time System instrument (Bio-Rad) and human TaqMan primer/probe gene expression assays that span exon-exon junctions (ThermoFisher Scientific). The threshold counts were normalized to the relative abundance of the TATA-binding protein (TBP) mRNA using the comparative CT method. Fold changes in gene expression are calculated as the fold change for the treatment groups versus control. To prepare protein extracts, cells treated with Dox, SB, or TGF-β2 were lysed using M-PER extraction buffer with the HALT phosphatase/protease inhibitor cocktail (both by ThermoFisher Scientific) on ice for 15 min. Protein concentrations in prepared extracts were quantified with BCA assay kit (ThermoFisher Scientific). Samples were denatured and reduced using 4X Laemmli sample buffer (Bio-Rad) with β-mercaptoethanol. Equivalent amounts of protein were loaded onto a gradient 4–15% TGX gel (Bio-Rad) and run at 125 V for 60–90 min in 1X Tris/Glycine/SDS buffer (Bio-Rad). The resolved proteins were transferred to a low fluorescence PVDF membrane using the semi-dry Trans-Blot Turbo transfer system (Bio-Rad). Membranes were blocked with a 1:1 ratio of SeaBlock (ThermoFisher Scientific) and TBS pH 7.4 and incubated with primary antibodies in blocking buffer with 0.1% Tween-20 overnight shaking at 4 °C. Secondary antibodies with the fluorescent tags 800CW and 680LT (LI-COR Biosciences) were diluted in blocking buffer with 0.1% Tween-20. Membranes were imaged on the Odyssey imaging system using Image Studio software for image capture and analysis (LI-COR Biosciences). The amount of phosphorylated protein per lane was normalized to the total parent protein. The amount of total protein per lane was normalized to β-actin.

### RNA sequencing and chromatin immunoprecipitation

To evaluate the effects of Dox and TGF-β2 on endothelial transcriptomes, total RNA was isolated from cultured cells using *mir*Vana RNA Isolation Kit (ThermoFisher). Endothelial cells were treated according to Dox treatment or Dox washout protocols. The effects of TGF-β2 were examined in separate experiments. Specifically, HCMVEC were starved for 4 h and treated with 0.3 ng/ml TGF-β2 for 16 h. RNA sequencing in the experiments with Dox and TGF-β2 was performed by the Novogene Corporation and Children’s Mercy Hospital Genomic facility, respectively. One microgram of RNA isolated from cultured cells was converted into cDNA libraries and prepared for sequencing using the TruSeq RNA sample preparation kit (Illumina). High throughput RNA-seq was performed by paired-end (2 × 101) deep sequencing coverage to an average depth of ~ 50 million reads with > 89% of bases above Q30 using Illumina’s TruSeq technology on the Illumina HiSeq1500. The resulting base calling (.bcl) files were converted to FASTQ files using Illumina’s bcl2fastq v2.17.1.14 software.

Chromatin immunoprecipitation (ChIP) was performed using the SimpleChIP enzymatic chromatin IP kit (Cell Signaling Technology, #9003) according to the manufacturer instructions. Briefly, cellular proteins were cross-linked to DNA with 1% formaldehyde and collected into PBS with supplied protease inhibitors. Chromatin digestion was performed with micrococcal nuclease followed by sonication to break nuclear membrane. An aliquot of chromatin was used to purify DNA and perform agarose gel electrophoresis in order to determine quality and quantity of digested DNA. The rest of the chromatin was incubated with anti-Smad3 antibody, and the antibody-DNA complex was pulled down with the supplied protein G magnetic beads and incubated with proteinase K to reverse crosslinking. The purified DNA was then used for ChIP-PCR and ChIP sequencing. The primer/probe TaqMan assay for ChIP-PCR was designed for the critical Smad3 binding site in the proximal TAGLN promoter, described in [42], using Primer Express software (ThermoFisher): forward primer, GCGGCAGCCCTTTAAACC; reverse primer, GCAGGAAGGAGTGAAGACTTGTG; probe, TCACCCAGCCAGCG. Real-time PCR was performed as described earlier [21]. In the ChIP seq workflow, DNA library preparation and sequencing were performed by the Novogene Corporation.

### Bioinformatic analysis

Partek Flow (version 12.4.3) analytical software suite by Illumina was used to analyze both transcriptomic and ChIP sequencing data. FASTQ files generated from ChIP sequencing were aligned by Burrow-Wheeler aligner for longer reads (BWA-MEM) to the human reference genome hg38. After filtering out duplicates, we used the Model-based Analysis of ChIP-seq (MACS) algorithm to identify transcription factor binding sites in control and Dox treated samples. Significant regions were called based on q-value (minimum FDR) set to 0.05. Filtered peaks within the transcriptional start site (TSS) region, with upstream and downstream limits set 2000 and 500 bp, respectively, were annotated according to Ensembl 112 release. Sets of genes that had significant peaks within the TSS region, were further examined using Gene Set Enrichment Analysis (GSEA) to identify the “biological processes” and “molecular functions” gene ontology terms. Additionally, the vertebrates motif database was used to search for known motifs with sequence quality of ≥ 0.7. We further compared gene counts with identified motifs in the immunoprecipitated Dox and control samples that were normalized using fragments per kilobase per million (FPKM). Poisson regression was used for differential analysis, and only genes with a false discovery rate (FDR) ≤ 0.05 after Benjamini correction for multiple testing were included in the final pathway analysis using Qiagen Ingenuity Pathway Analysis (IPA) [43].

To analyze the RNA seq data, reads with Phred quality score ≥ 35 and at least 100 bp long were used in downstream analysis. Alignment was performed using the STAR 2.7.8a aligner against the human hg38 reference genome and Ensembl transcripts (release 109) index. After alignment, low-quality reads (mapping quality < 20), singletons, and unaligned reads were filtered out. For differential gene expression analysis in the TGF-β2 treatment protocol, transcripts per kilobase million (TPM) normalization was applied followed by a Poisson regression model with the Wald statistic for *p*-value calculation. Genes meeting the significance threshold of *p* < 0.05 (after Bonferroni correction for multiple testing) with a ≥ twofold change between groups were selected for further analysis. In the Dox treatment/washout experiments, differential expression of protein coding genes was analyzed after median ratio normalization using the DESeq2 method. Genes meeting the significance threshold of *p* < 0.05 (after Bonferroni correction for multiple testing) with a ≥ twofold change between groups were selected for further analysis. Differentially expressed genes identified in the TGF-β2 and Dox experiments were uploaded into QIAGEN Ingenuity Pathway Analysis (IPA) to examine the key canonical pathways and upstream regulators within the compared datasets. For the canonical pathway analysis, we examined molecules mapped to canonical pathways curated in the QIAGEN Knowledge Base. The association between the datasets and each canonical pathway was assessed using two key metrics, pathway involvement ratio (as a proportion of pathway related molecules in the dataset and the total number of molecules in that pathway), and statistical significance (as *p*-values determined by right-tailed Fisher’s Exact Test evaluating the probability that the observed association between the dataset and the pathway occurs by chance). Additionally, a *z*-score was derived from expression trends within the dataset to predict activity of specific pathways. IPA was also utilized to predict activity of upstream regulators, molecules that influence gene expression, transcription, or phosphorylation. Such predictions were based on the observed upregulated and downregulated transcripts within datasets with known regulatory relationships.

### Reporter plasmids and cell transfection

pMCS-Red Firefly luciferase and Renilla luciferase were purchased from ThermoFisher (catalog #16155 and #16153, respectively). CAGA_12_-luciferase reporter plasmid, described in [44], was a gift by Dr. Caroline Hill of the Francis Crick Institute. Tagln gene promoter (1140 bp) was cloned using human genomic DNA and the following set of primers: forward 5′-CACCGGTACCGTCCAGGGATCCCACTGTTAGTC-3′; and reverse 5′-CCTAAAGCTTAGGCTTCCTCAGGGCTCGC-3′. The promoter amplicon was inserted into the pMCS-Red Firefly luciferase plasmid between Kpn1 and HindIII sites using T4 ligase (ThermoFisher, catalog #15,224–017). The insert incorporated into the plasmid backbone was validated by sequencing. HUVEC were plated in 12-well plates and transfected with the described reporter plasmids using X-fect transfection reagent (Takara Bio, #631318). Luciferase activities were measured using Dual-Glo Luciferase Assay System (Promega, #E2920) according to manufacturer’s instructions. Luciferase activity is presented as Firefly/Renilla luciferase ratio.

### Immunostaining and fluorescent microscopy

For immunohistochemistry staining, mice were injected intraperitoneally with 10U/ml heparin, anesthetized with 3% isoflurane mixed with 1L/min O_2_ via a facemask, and perfused via left ventricle with 10U/ml heparin-saline solution. The hearts were then excised incubated in 4% paraformaldehyde (PFA) overnight at 4 °C and processed for immunohistochemistry staining as described in [21]. To evaluate accumulation of Dox in cardiac endothelial cells, excised hearts were immediately cannulated and briefly perfused via aorta at a rate of 1 ml/min with PBS containing 10 U/ml heparin followed by PBS containing 4% PFA. Hearts were then immediately flash-frozen in the OCT compound cooled with isopentane on dry ice. Cardiac sections were mounted with Fluoromount-G (Southern Biotech) containing 2 uM Hoechst 33,342 and imaged immediately. Nuclear accumulation of Dox in live endothelial cultures was examined after staining of nuclei using 2 µM Hoechst 33,342 for 10 min in EGM2 culture media. For immunocytochemistry staining, treated HUVEC cultures were fixed in 4% PFA and blocked/permeablized using 3% BSA containing 0.3% Triton X-100. Cells were then incubated overnight at 4 °C with antibodies against transgelin, fibronectin, and CD31. Following incubation with the fluorophore-conjugated secondary antibodies, cell nuclei were stained using Hoechst 33342. For filamentous actin (F-actin) staining, 1 U/ml Texas Red X-conjugated phalloidin was added to the Hoechst 33342 solution for a 10-min room temperature incubation. Image acquisition and analysis of fluorescent images were performed using Cytation 5 imaging system with Gen5 version 3.14 Image Prime software (Agilent Technologies).

### Endothelial barrier function assay

Endothelial monolayer permeability assay was performed using Transwell 24-well plate with permeable 0.4 µm polyester membrane inserts (Corning, 3470). Twenty five thousand cells were seeded per well and cultured for 3 days to facilitate formation of a confluent monolayer. Cultures were then treated with Dox and/or SB before removing Dox from culture media for the 48-h washout period. Fluorophore conjugated 10,000 and 70,000 MW dextrans (200 µg/ml each) were added into the insert chamber. The endothelial barrier function was evaluated by measuring concentrations of the permeability probes in the lower chamber over time.

### Statistical analysis

ChIP sequencing and transcriptomic data analyses are described in Sect. "RNA sequencing and chromatin immunoprecipitation". Statistical analyses of other datasets were performed using GraphPad Prism (version 9.1.2) software package. Unpaired *t*-test was used to perform comparisons between two experimental groups. When more than two experimental groups within a dataset were to be compared, one- or two-way analysis of variance (ANOVA) followed by either Sidak’s or Tukey’s correction tests were utilized, as suggested by statistical software. Effect size was calculated in selected experiments using Cohen’s *d* parameter. Results are expressed as mean ± standard deviation. A *p* < 0.05 was considered statistically significant.

## Results

### Enhanced activation of the canonical TGF-β/Smad3 pathway in endothelial cells after completion of Dox therapy

**Fig. 1:**
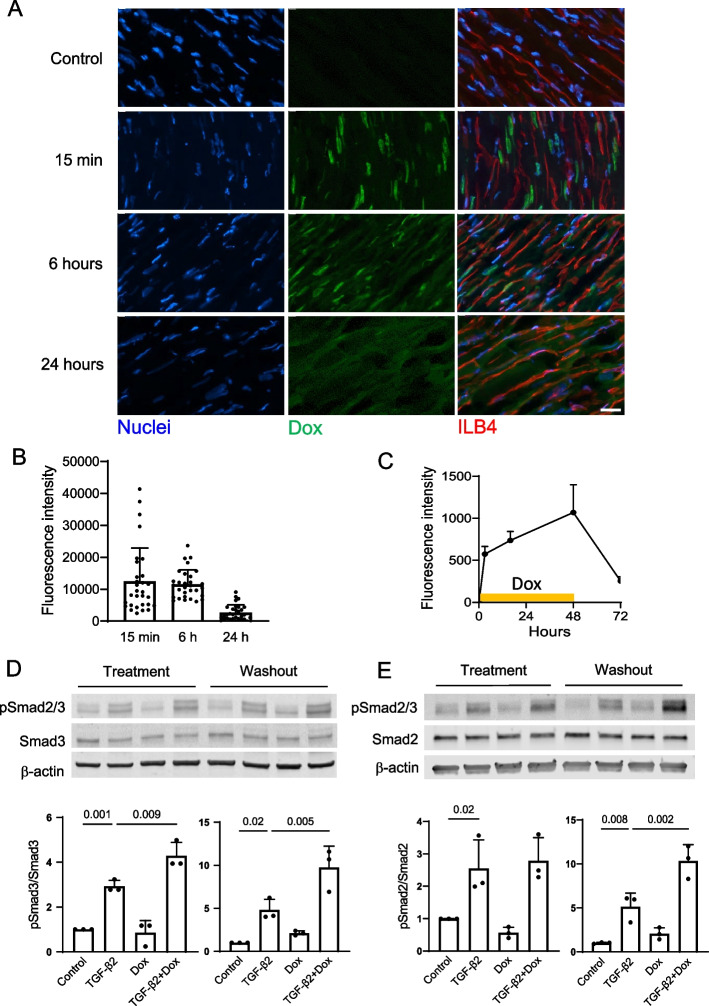
Enhanced Smad2/3 phosphorylation in endothelial cells upon Dox washout. **A** Accumulation of Dox in nuclei of cardiac endothelial cells of treated mice after its intravenous injection of 5 mg/kg. Mice were sacrificed at the specified time points after the injection (*n* = 3 per group). Control mice (*n* = 2) did not receive doxorubicin. Intrinsic fluorescence of doxorubicin was pseudocolored green. Nuclei were stained with 2 μM Hoechst 33,342 while endothelial cells were identified using the ILB4 conjugate. Scale bar, 20 μm. **B** Analysis of Dox accumulation in mouse cardiac endothelial nuclei after the injection. Fluorescence intensity of 10 endothelial cell nuclei per heart were quantified. **C** Analysis of Dox (100 nM) accumulation in cultured HUVEC nuclei during treatment and washout. Intrinsic fluorescence of the Dox molecule was quantified in nuclei visualized using the Hoechst 33,342 (2 μM) staining of live cultures at specified time points. Dox was removed from media after the 48-h treatment period. **D** Smad3 and (**E**) Smad2 phosphorylation responses to 0.3 ng/ml TGF-β2 after 48-h Dox (16 nM) treatment and 48-h washout. Cultures were starved in growth factor free endothelial basal media for 4 h before the one-hour TGF-β2 incubation. Representative immunoblots and analysis of n = 3 independent experiments are shown. pSmad2/3, phospho-Smad2/3. pSmad3 and pSmad2 band intensities are presented as phospho- to corresponding total protein ratios (*n* = 3 biological replicates for both pSmad2 and pSmad3 experiments). The* p* values for the one-way ANOVA followed by Sidak correction method are shown

We examined Dox accumulation in murine cardiac endothelial cells after its intravenous injection, an administration route that is used clinically. Intrinsic fluorescence of the Dox molecule was utilized to evaluate accumulation of the drug in endothelial cells that were labeled using fluorophore conjugated lectin ILB4 (Fig. 1A). At 15 min after the injection, Dox primarily accumulated in anuclear cells within the microvascular lumen, most likely erythrocytes, and a subset of endothelial cell nuclei. The drug consistently accumulated in endothelial nuclei after 6 h and its levels markedly declined at the 24-h time point after the injection (Fig. 1B). Despite transient exposure, endothelial dysfunction in mouse hearts persisted for weeks after Dox injections [21]. To examine the nature of sustained endothelial dysfunction, we utilized the Dox treatment followed by washout protocol to model transient exposure to Dox in treated animals. As a known DNA intercalating agent, Dox accumulated in nuclei of cultured endothelial cells during the 48-h treatment period (Fig. 1C). Media replacement to start the washout period led to a significant decline in nuclear Dox levels validating the endothelial culture Dox treatment/washout protocol as a model of transient accumulation of the drug after the intravenous injection.

We have previously reported increased cardiac and endothelial production of the selected TGF-β family ligands and activity of the canonical TGF-β pathway during Dox treatment [21, 27], and sought to examine activity of the pathway after Dox removal. Endothelial Smad3 phosphorylation response to TGF-β2 was elevated during Dox treatment and even further enhanced upon its washout (Fig. 1D). Smad2 phosphorylation was not increased by Dox during treatment but, similarly to Smad3, was markedly enhanced during its washout (Fig. 1E). We further utilized CAGA_12_-luciferase, a sensitive TGF-β/Smad3 pathway reporter containing 12 copies of the Smad3 binding element [44], in our experiments. Activity of the reporter was responsive to TGF-β2 (Suppl. Figure 4 A) and elevated during Dox washout (Fig. 6C). Elevated phosphorylation and transcriptional activity of Smad2/3 transcription factors suggested that increased activity of the canonical TGF-β pathway in endothelial cells persisted after Dox washout.

**Fig. 2:**
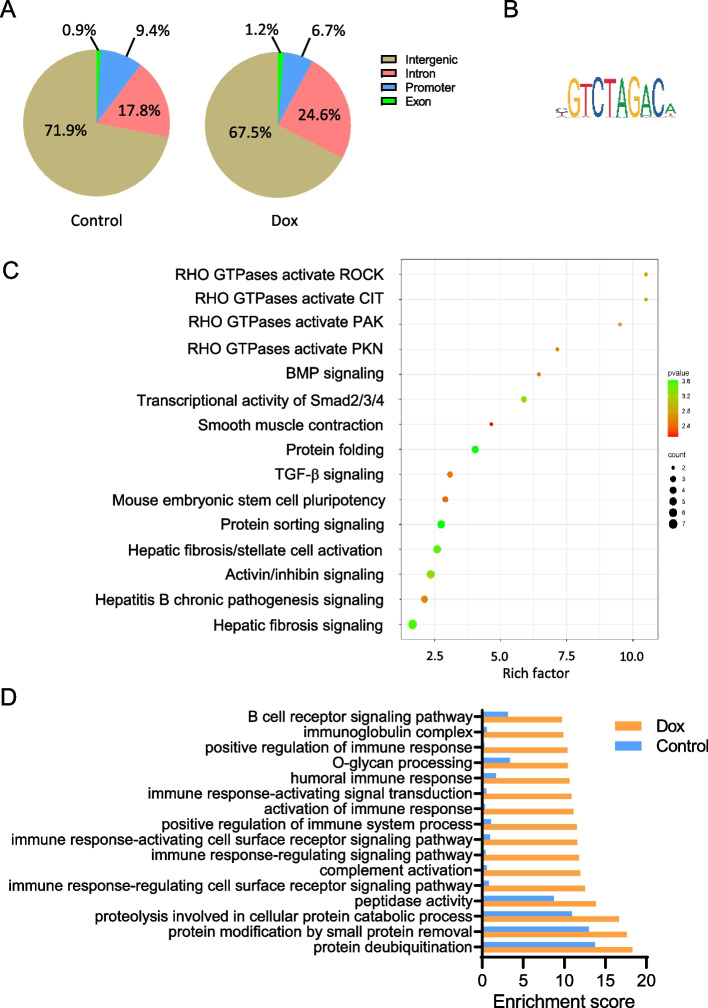
Analysis of genome-wide Smad3 binding in endothelial cells upon Dox washout. ChIP sequencing using the Smad3 antibody was performed on the HUVEC cultures treated with 16 nM Dox followed by 48-h washout. **A** Gene regions for the Smad3 binding sites were determined using peak calling and annotation of the peaks in Partek Flow. **B** The most enriched transcription factor motif in the identified Smad3 binding sites. **C** Assigned peaks for Smad3 binding sites in the TSS regions of the immunoprecipitated control and Dox samples were used to create gene lists that were analyzed using the IPA to determine the canonical pathways enriched in Dox treated versus control cells. ROCK, Rho-associated protein kinase; CIT, citron kinase; PAK, p21-activated kinase; PKN, protein kinase N; BMP, bone morphogenetic protein. Statistical significance is presented as -log (*p* value). **D** The same gene lists were analyzed with Gene Sets Enrichment Analysis (GSEA) using biological processes and molecular function aspects of gene ontology (GO) terms

These results prompted us to examine genome wide Smad3 binding sites in the treated endothelial cells. ChIP seq experiments with Smad3 antibody demonstrated increased Smad3 binding in the intronic gene regions after Dox washout, as compared to control, at the expense of intergenic and promoter regions (Fig. 2A). As expected, the canonical Smad3 binding motif CAGA/GTCT was the most predominant at Smad3 sites in both control and Dox treated endothelial cells (Fig. 2B). In our further analysis, we focused on the Smad3 binding sites within the promoter regions because these could be readily assigned to specific genes and are likely to contribute to regulation of their transcription. Initially, these gene sets were uploaded into IPA to examine canonical pathways that were overrepresented in Dox treated versus control cells. The results of this analysis, presented in Fig. 2C, suggest enrichment in pathways involved in smooth muscle contraction/cytoskeletal and fibrotic remodeling, TGF-β, BMP, activin/inhibin and Smad2/3/4 signaling pathways. The same gene sets underwent the GSEA to determine the most represented gene ontology terms in both control and Dox treated cells, including specific molecular functions and biological processes (Fig. 2D). Genes involved in immune/inflammatory and ubiquitination/proteolysis processes were highly enriched in Dox treated endothelial cells, as compared to control. The results of this analysis further reinforce our earlier data on the Smad3 dependent upregulation of the inflammatory pathways in Dox treated cardiac endothelial cells [21].

**Fig. 3:**
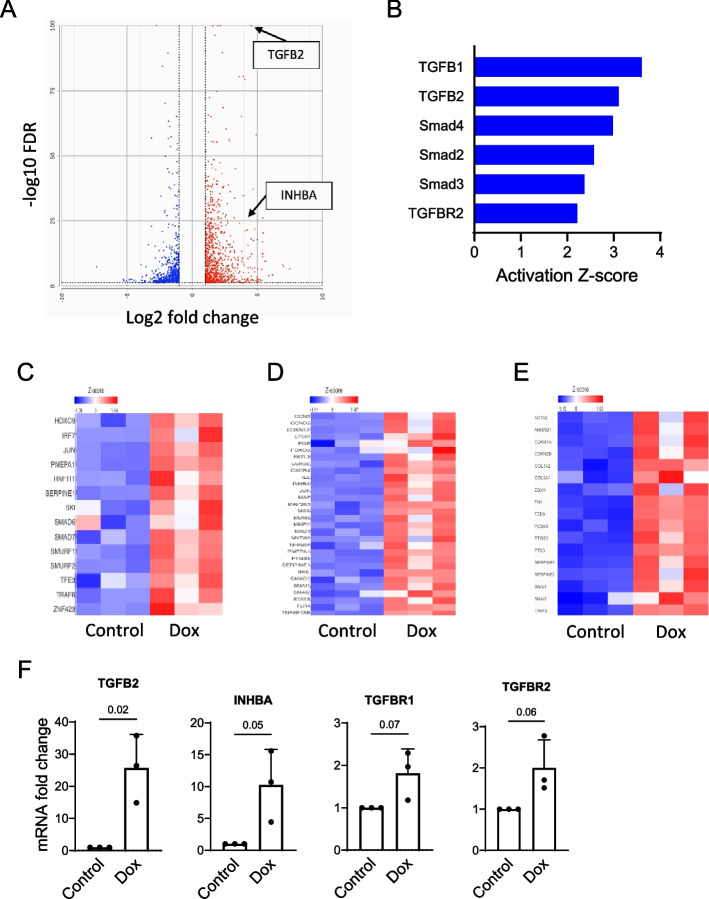
Enhanced activities of the TGF-β and activin pathways in endothelial cells during Dox washout. HUVEC were treated with Dox (16 nM) for 48 h followed by the 48-h washout period, and total RNA was isolated for transcriptomic analysis (*n* = 3 independent experiments per treatment group). **A** A volcano plot presenting significantly different differentially expressed protein coding genes in Dox versus control samples. Positions of the *Tgfb2* and *Inhba* transcripts are indicated with arrows. **B** Significantly different differentially expressed protein-coding gene lists were analyzed with IPA to identify the upstream regulators related to the TGF-β/activin pathways and their predicted degrees of activation using Z-scores. **C**, **D**, and **E** Heatmaps presenting Z-scores for TGF-β and activin pathways related transcripts, and Smad3 target genes, respectively. **F** Validation of the transcriptomic results using qPCR analysis of expression of selected TGF-β/activin pathways related transcripts (*n* = 3 biological replicates). *Tgfb2*, transforming growth factor beta 2; *Inhba*, inhibin subunit beta A; *Tgfbr1*, transforming growth factor beta receptor 1; *Tgfbr2*, transforming growth factor beta receptor 2. The* p* values for the unpaired two-tailed *t*-test are shown

We then performed transcriptomic analysis to further probe endothelial responses to Dox during both treatment and washout. Dox treated versus control (untreated) transcriptomes were compared to identify differentially expressed genes both at the end of the treatment period and Dox washout (Suppl. Figure 1 A and Fig. 3A, respectively). Among them were *Tgfb2*, *Inhba*, and *Inhbb* transcripts encoding the TGF-β superfamily ligands that act in the activin and TGF-β pathways. Importantly, expression of *Tgfb2* and *Inhba* genes was further elevated upon Dox removal. Increased expression of these transcripts by Dox was confirmed using qPCR (Fig. 3D and Suppl. Figure 1 F). The groups of the differentially expressed genes were further probed using Ingenuity Pathway Analysis (IPA). The analysis predicted Smad2/3/4 to be among the top upstream regulators that significantly contributed to the differential gene expression pattern during both Dox treatment and washout (Fig. 3B and Suppl. Figure 1B). The performed IPA analysis clearly corroborated the results of the Smad2/3 phosphorylation and CAGA_12_-luciferase reporter activity during both Dox treatment and washout. Smad2/3 transcription factors function in both TGF-β and activin canonical pathways. To illustrate this relationship, the increased expression of the Smad3 transcriptional target genes (Fig. 3E and Suppl. Figure 1E) coincides with increased expression of the TGF-β and activin related transcripts (Fig. 3C and D, and Suppl. Figure 1 C and D, respectively).

### Activation of mesenchymal transcription in endothelial cells after completion of Dox therapy

As Dox treated cells display both increased production of TGF-β2 and augmented endothelial Smad3 phosphorylation response to it we examined the effects of TGF-β2 on endothelial cells. Specifically, we focused on mesenchymal reprogramming, fibrotic response, and cytoskeletal remodeling, as suggested by the enriched in Smad3 binding canonical pathways in Dox treated endothelial cells (Fig. 2C). To evaluate the effects of the TGF-β2 treatments, we compared transcriptomes of TGF-β2 treated versus control endothelial cells. As shown in Suppl. Figure 2 A and 2B, epithelial-to-mesenchymal transition (EMT) and fibrotic pathways were significantly activated in the TGF-β2 treated cultures. The role of Smad3 in these endothelial responses to TGF-β2 was assessed in Smad3 deficient cells that were described in detail in our previous publication [21]. Pairwise comparison of Smad3 shRNA + TGF-β2 with scrambled shRNA + TGF-β2 revealed dramatic downregulation of EMT and profibrotic pathways in Smad3 deficient cells (Suppl. Figure 2 A and 2B). The transcriptomic analysis was further supported by the results of the qPCR experiments, where Smad3 knockdown significantly decreased the abundance of the elevated by TGF-β2 transcripts encoding extracellular matrix proteins (*Col1a1, Col1a2, Col3a1, and Fn1*), smooth muscle cell marker (*Acta2*), and an EMT inducing transcription factor (*Snai2*), as shown in Suppl. Figure 2C.

**Fig. 4:**
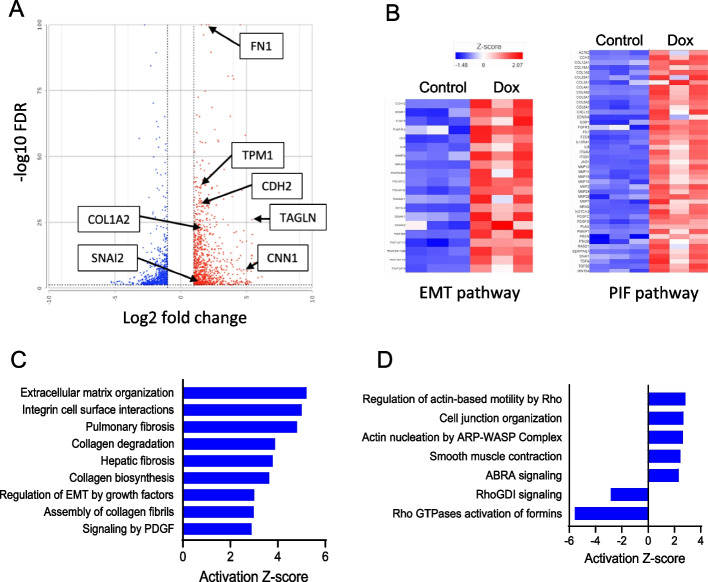
Transcriptional analysis of mesenchymal activation in endothelial cells upon Dox washout. HUVEC were treated with Dox (16 nM) for 48 h followed by the 48-h washout period, and total RNA was isolated for transcriptomic analysis (*n* = 3 biological replicates per treatment group). **A** A volcano plot presenting significantly different differentially expressed protein coding genes in Dox versus control samples. Positions of the mesenchymal transcripts are indicated with arrows. **B** Heatmaps presenting Z-scores for the epithelial-to-mesenchymal (EMT) and pulmonary idiopathic fibrosis (PIF) pathways related transcripts. **C** and (**D**) Significantly different differentially expressed protein-coding gene lists were analyzed with IPA to identify the upstream regulators related to the fibroblastic/EMT and smooth muscle/cytoskeletal regulation pathways, respectively, and their predicted degrees of activation using Z-scores

We then examined if Dox promotes mesenchymal activation in treated endothelial cells that did not undergo Dox washout. While comparing significantly upregulated differentially expressed transcripts in treated and control conditions it was determined that Dox treatment indeed activates the EMT and pulmonary idiopathic fibrosis (PIF) pathways (Suppl. Figure 3 A and 3B). Abundance of selected transcripts and proteins was validated using qPCR (Suppl. Figure 5 A and 5B) and immunoblotting (Suppl. Figure 5A-C), respectively. Importantly, mesenchymal activation not only persisted but was further enhanced upon Dox washout in endothelial cells (Fig. 4A and 4B). Upon further analysis, it was determined that the upregulated mesenchymal transcripts can be grouped into fibroblastic/extracellular matrix and smooth muscle/cytoskeletal remodeling categories. Viewed from this perspective, we identified the canonical pathways related to fibrotic and cytoskeletal remodeling both during Dox treatment (Suppl. Figure 3 C and 3D) and washout (Fig. 4C and 4D).

**Fig. 5:**
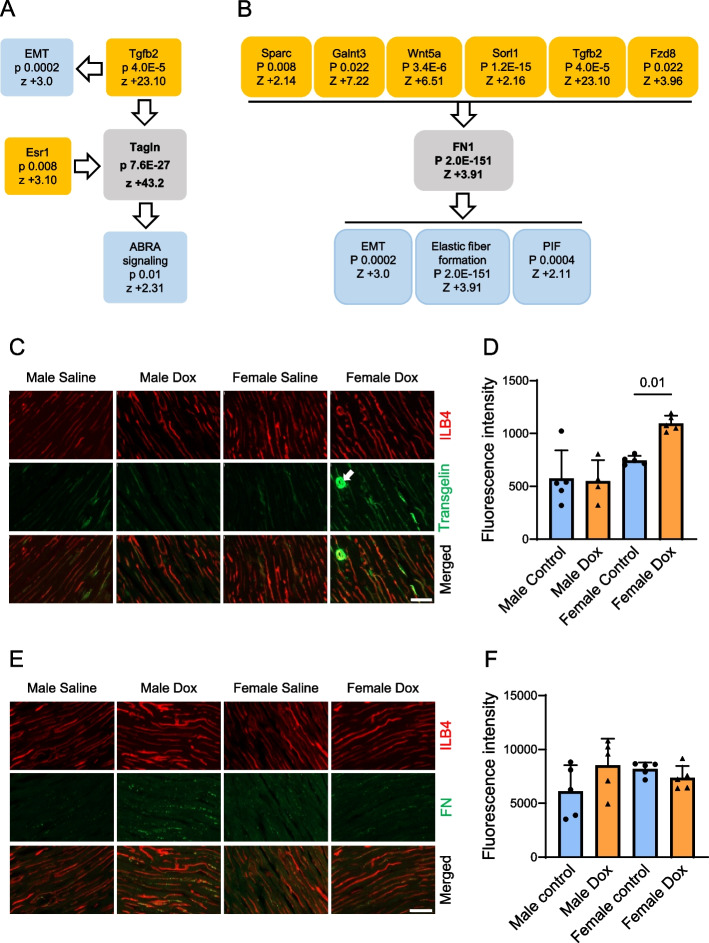
Fibroblastic (fibronectin) and smooth muscle (transgelin) lineage specific markers after completion of Dox treatments. **A** and (**B**) HUVEC were treated with Dox (16 nM) for 48 h followed by the 48-h washout period, and total RNA was isolated for transcriptomic analysis (*n* = 3 biological replicates per treatment group). IPA generated regulatory network analysis for a fibroblastic (*Fn1*) and a smooth muscle (*Tagln*) genes (shown in grey boxes), respectively. For each of these genes, the lists of upstream regulators that are predicted to have contributed to their upregulation (orange boxes, which present *p* values of overlap and Z-scores), and downstream signaling pathways (blue boxes, which also present *p* values of overlap and Z-scores) are shown. **C** to (**F**) Male (*n* = 9) and female (*n* = 10) mice received four i.v. injections of Dox or saline over a period of two weeks and sacrificed three weeks after the last injection for the immunohistochemical analyses. Endothelial cells were visualized using isolectin ILB4 staining. **C** presents expression of a smooth muscle protein marker, transgelin, in cardiac microvascular endothelial cells of male and female mice. A cardiac arteriole featuring smooth muscle media layer is indicated with the arrow. Scale bar, 50 µm. **E** shows expression of a fibroblastic protein marker, (fibronectin, FN), in cardiac microvascular endothelial cells of male and female mice. Scale bar, 50 µm. Analyses of transgelin and FN expression are shown in (**D**) and (**F**), respectively. *n* = 4 to 5 animals per treatment group. The* p* values for the one-way ANOVA followed by Sidak correction method are shown

Our further analysis focused on the selected transcripts of fibroblastic (*Fn1*) and smooth muscle (*Tagln*) lineages to build networks presenting the upstream regulators that are predicted to have contributed to their increased expression, and downstream pathways these transcripts may be regulating (Fig. 5A and 5B, respectively). Notably, TGF-β2 is predicted to be the strongest upstream regulator for both transcripts. These results suggest sustained mesenchymal reprogramming that persists in treated endothelial cells upon Dox washout and is predicted to promote fibrotic and cytoskeletal remodeling. To evaluate the effects of the drug on reprograming of cardiac microvascular endothelium in vivo, we performed four intravenous injections of Dox to male and female mice and harvested cardiac tissue three weeks after its last injection. Increased endothelial expression of the smooth muscle protein marker transgelin, encoded by the *Tagln* gene, was observed at that time point in female but not male cardiac endothelial cells (Fig. 5C and 5D). As shown in Fig. 5A, the IPA analysis of upstream regulators leading to upregulated *Tagln* expression included Esr1, a transcript that encodes the estrogen receptor alpha (ERα) protein. The predicted by this analysis role of estrogen signaling may have contributed to sex dimorphism in transgelin expression in this model. On the other hand, abundance of fibronectin, a fibroblastic protein marker was moderately elevated, with large Cohen’s *d* effect size of 1.0, in microvascular endothelial cells and pericapillary area in male but not female mice (Fig. 5E and 5 F). These results suggest that sustained mesenchymal reprogramming occurs in cardiac microvasculature after completion of Dox treatment.

### ALK4/5/7 inhibitor blunts mesenchymal activation by Dox in endothelial cells

**Fig. 6:**
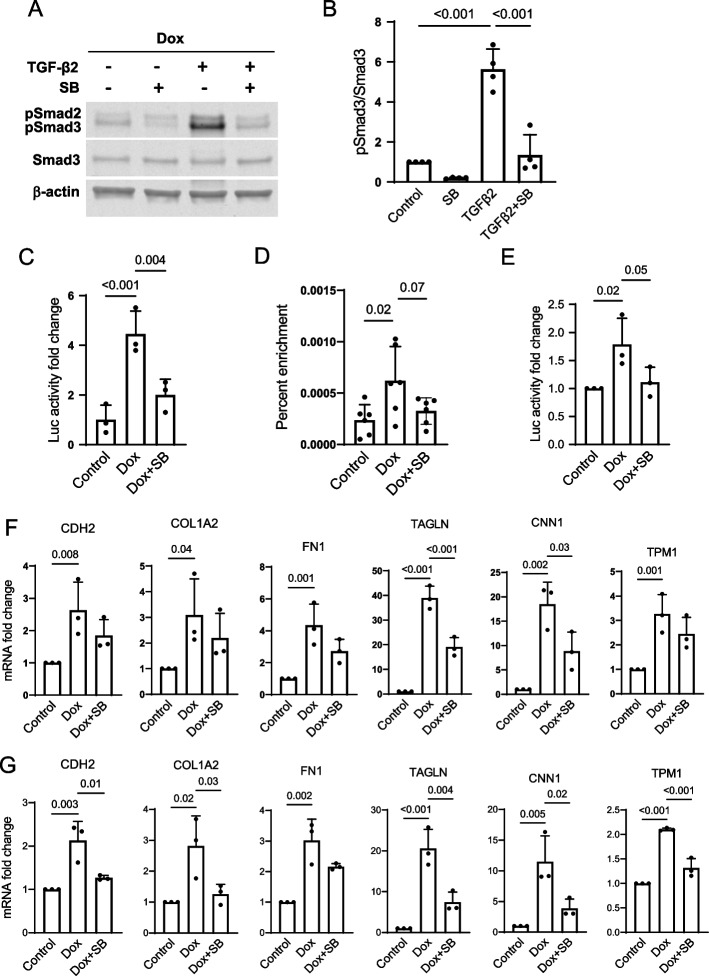
ALK4/5/7 inhibitor suppresses the TGF-β pathway and expression of mesenchymal genes in endothelial cells. HUVEC were treated with Dox (16 nM) for 48 h followed by the 48-h Dox washout period. SB (1 µM) was present either during Dox treatment or during Dox washout only. **A** Cells were starved at the end of the washout period and treated with 0.3 ng/ml TGF-β2 for 60 min. SB (1 µM) was present during starving and TGF-β2 treatment periods. **B** Smad3 phosphorylation response to TGF-β2 is shown as a pSmad3/Smad3 ratio (*n* = 4 biological replicates). **C** Transfection with CAGA_12_-luciferase and Renilla luciferase plasmids was performed after 48-h treatment with Dox and/or SB; the transfected cultures were incubated for the 48-h washout period. Data are presented as CAGA_12_-luciferase/Renilla luciferase activity ratio and normalized to the control (*n* = 3 biological replicates). **D** Cells were collected after 48-h treatment with Dox and/or SB followed by no-treatment washout and processed according to the ChIP protocol with the Smad3 antibody. The input and immunoprecipitated (IPed) DNA samples were used in qPCR reaction and the results are presented as percent IPed/input enrichment ratio (*n* = 6 biological replicates). **E** HUVEC were treated with Dox and/or SB and transfected with the *Tagln* promoter-luciferase and Renilla luciferase plasmids. Cells were lysed for luciferase activity measurements after the 48-h no-treatment washout period. Data are presented as *Tagln* promoter-luciferase/Renilla luciferase activity ratio and normalized to control (*n* = 3 biological replicates). **F** HUVEC were treated with Dox and/or SB followed by no-treatment washout, total RNA was isolated and used to quantify expression of the selected mesenchymal transcripts (*n* = 3 biological replicates). **G** HUVEC were treated with Dox while SB was present during the 48-h washout period only (*n* = 3 biological replicates). Total RNA was isolated after the washout period and used to quantify expression of the selected mesenchymal transcripts. The* p* values for the one-way ANOVA followed by Sidak correction method are shown

Given a principal role the TGF-β/activin/Smad2/3 pathways are predicted to play in the described endothelial reprogramming by Dox, we utilized a prototypical and highly selective inhibitor SB431542 (SB) that blocks Smad2/3 phosphorylation by the TGF-β and activin ligands acting on their cognate ALK4/5 receptors [41]. It abolished the Smad2/3 phosphorylation response to TGF-β2 in endothelial cells during Dox washout (Fig. 6A and 6B). In addition, we utilized the CAGA_12_-luciferase reporter plasmid to demonstrate that SB suppresses Smad3 transcriptional activity that was upregulated in endothelial cells upon Dox washout (Fig. 6C). In the Dox treatment protocol, SB prevented upregulation of fibroblastic (*Cdh2, Col1a2, Fn1*) and smooth muscle lineage (*Tagln, Cnn1, Tpm1*) transcripts (Suppl. Figure 5 A and B). During Dox washout, most of the studied mesenchymal transcripts remained elevated while expression of *Tagln* and *Cnn1* genes was further upregulated (Fig. 6F). When SB was present concurrently with Dox but not during Dox washout there was a modest (*Cdh2, Col1a2, Fn1*, and *Tpm1*) or significant (*Tagln, Cnn1*) decrease in expression of mesenchymal genes. On the other hand, treatment with SB during Dox washout only effectively reduced expression of all studied transcripts suggesting a role of Smad2/3 activity in the sustained mesenchymal activation upon Dox washout (Fig. 6G). The SB effect size was calculated in these experiments by comparing Dox versus Dox + SB treatment groups. As presented in Suppl. Table 1, the effect size for concurrent only and washout only treatment with SB was medium-to-large and large, respectively.

Of all mesenchymal transcripts, Tagln was most highly upregulated, both during Dox treatment and, especially, during its washout. Activation of the *Tagln* gene causes alterations in cytoskeletal organization, including filamentous actin (F-actin) network, and is required for cell differentiation into mesenchymal lineages [45]. Therefore, we selected the *Tagln* gene to further examine the mechanisms of its sustained upregulation after Dox removal. Smad3 binding at the proximal *Tagln* promoter site that activates its transcription [42] was increased, as shown in our TGF-β2 treatment and Dox washout experiments (Suppl. Figure 4B and Fig. 6D, respectively). In addition, we observed a strong trend towards reduced Smad3 binding in the Dox washout experiments with SB that was present during Dox treatment only. To further study the processes behind increased expression of *Tagln* by Dox we utilized the *Tagln* promoter-luciferase reporter plasmid. Similarly to several other cell types [42], activity of the promoter was increased by TGF-β2 in endothelial cells (Suppl. Figure 4 C). Likewise, activity of the *Tagln* promoter was elevated during Dox washout, and concurrent treatment with SB reduced it to the level of untreated control (Fig. 6E). Collectively, these results suggest that elevated *Tagln* expression in this model is due to increased Smad3 promoter binding and its enhanced transcriptional activity.

**Fig. 7:**
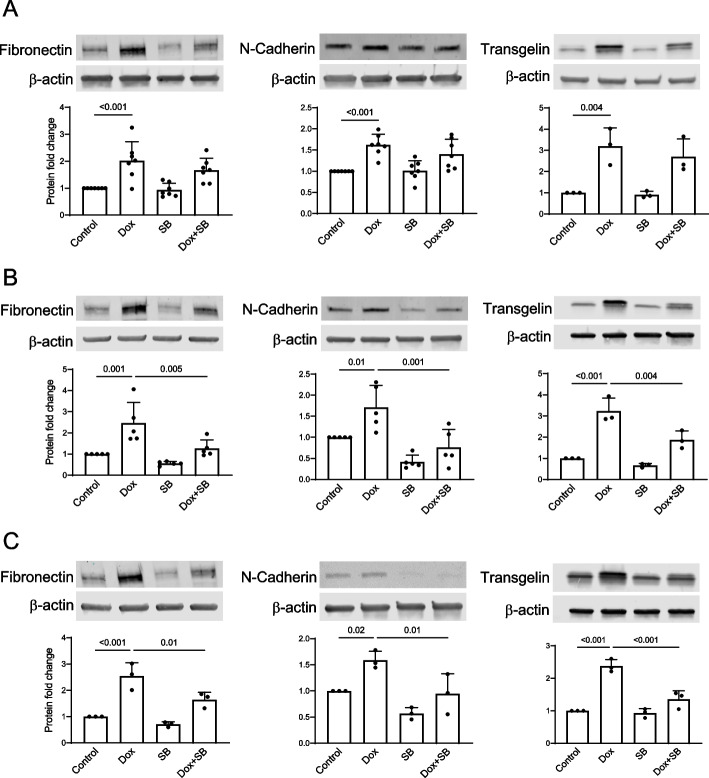
ALK4/5/7 inhibitor suppresses increased endothelial expression of mesenchymal protein markers upon Dox washout. HUVEC were treated with Dox (16 nM) for 48 h followed by the 48-h Dox washout period. SB (1 µM) was present either during Dox treatment only (**A**), washout only (**B**), or throughout the Dox treatment/washout cycle (**C**). Protein extracts were prepared at the end of the washout period and processed according to the immunoblotting protocol. Representative blots and expression analyses are shown for each mesenchymal protein marker (*n* = 3 to 7 biological replicates). The* p* values for the one-way ANOVA followed by Sidak correction method are shown

**Fig. 8:**
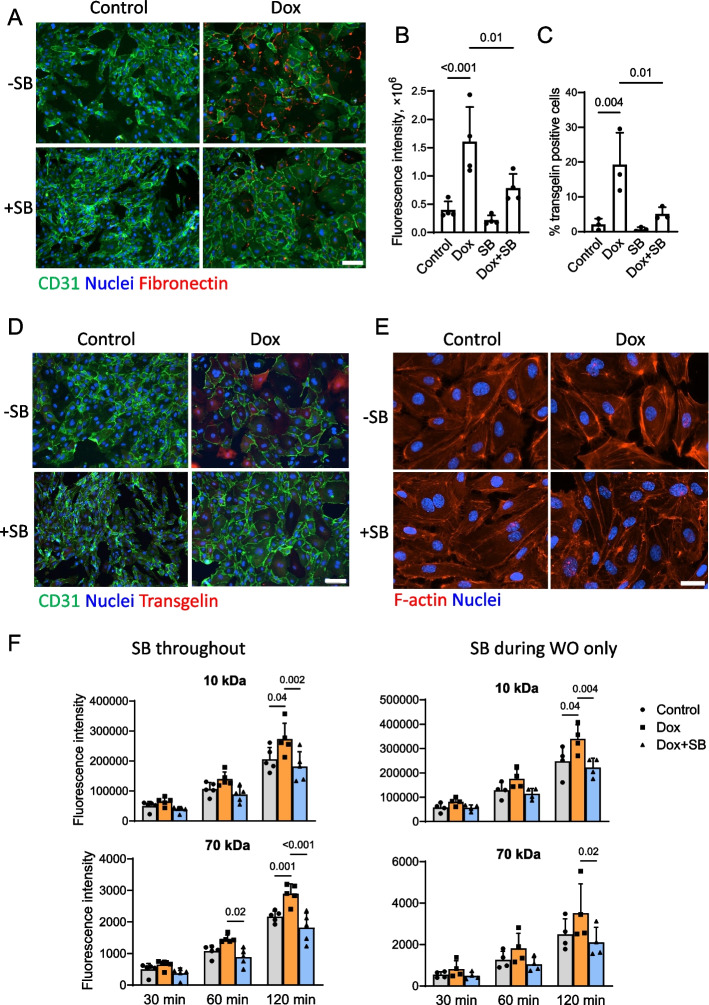
ALK4/5/7 inhibitor decreases mesenchymal protein expression and enhanced endothelial monolayer permeability upon Dox washout. HUVEC were treated with Dox (16 nM) for 48 h followed by the 48-h Dox washout period. SB (1 µM) was present throughout the Dox treatment/washout cycle. **A** HUVEC cultures were fixed at the end of the Dox washout period and processed according to the immunostaining protocols. Representative images of HUVEC cultures stained for fibronectin. Scale bar, 100 µm. **B** Fluorescence intensity analysis of fibronectin expression (*n* = 4 biological replicates). **C** Analysis of transgelin expression in treated endothelial cells (*n* = 3 biological replicates). The* p* values for the one-way ANOVA followed by the Sidak correction method are shown. **D** Representative images of HUVEC cultures stained for transgelin. Scale bar, 100 µm. **E** HUVEC cultures were fixed at the end of the Dox washout period and incubated with Texas Red X-conjugated phalloidin and Hoechst 33,342 to stain F-actin and nuclei, respectively (*n* = 3 biological replicates). Scale bar, 80 µm. **F** HUVEC were plated onto the permeable membrane inserts, cultured to form a confluent monolayer, and then treated with Dox and/or SB, either during both Dox treatment and its washout (SB throughout), or during Dox washout (WO) only. Fluorophore-conjugated dextrans (MW 10 and 70 kDa) were added to the insert chamber at the end of the Dox washout period and fluorescent intensities of the dextrans leaked into the lower chamber were measured at the indicated time points. *n* = 4 to 5 biological replicates. The *p* values for the two-way ANOVA followed by the Tukey correction method are shown

Expression of mesenchymal protein markers largely followed the trends described for their transcripts. Specifically, with the Dox treatment protocol, expression of fibronectin and transgelin was increased by the drug, and concurrent treatment with SB decreased the levels of the studied proteins (Suppl. Figure 5 C). Increased expression of the mesenchymal marker proteins persisted during Dox washout in all experiments (Fig. 7A), and SB decreased their levels when present either during washout only or throughout the treatment/washout periods (Fig. 7B and C). The immunostaining data supported our immunoblotting results and emphasized additional important points. Specifically, fibronectin protein in the Dox washout experiments is found in the extracellular space, indicating not only increased expression but also secretion of the protein by the cultured endothelial cells that may contribute to remodeling of extracellular matrix in the vascular compartment (Fig. 8A and B). In addition, we observed that endothelial cells, identified using an endothelial marker CD31, expressed transgelin in Dox washout experiments (Fig. 8C and D). SB treatment once again reduced abundance of both of these mesenchymal protein markers during Dox washout.

### ALK4/5/7 inhibitor mitigates endothelial barrier dysfunction after completion of Dox therapy

Both ChIP seq and transcriptomic analyses predicted increased contractility and enhanced formation of the F-actin network in Dox treated endothelial cells that may lead to cytoskeletal and cell junction reorganization. We visualized F-actin filaments with phalloidin staining, as shown in Fig. 8E. Formation of central F-actin fibers that spanned across cell bodies was enhanced upon Dox washout. Centrally located F-actin network was not evident but peripheral fibers underlying the cell membrane remained intact in cultures treated with Dox + SB. We then determined if the described cytoskeletal changes have functional consequences, specifically, for endothelial monolayer permeability. Endothelial cells were plated into a permeable cell culture insert, which served as an upper chamber in the permeability experiments, and allowed for a confluent monolayer to form. After the Dox treatment/washout period, fluorophore conjugated 10 kDa and 70 kDa dextrans were added into the inserts to evaluate permeability of the monolayers by measuring their concentrations in the lower chamber. Increased endothelial permeability for both tracers was observed in Dox treated monolayers and completely prevented by SB that was present throughout the Dox treatment/washout protocol (Fig. 8F). Importantly, SB was as effective when present during the washout period only indicating reversal of the endothelial permeability defect in endothelial cultures previously treated with Dox.

## Discussion

It has been firmly established in clinical and animal studies that cardiovascular dysfunction persists and further progresses after completion of anthracycline therapy [23, 24, 46]. Considerable effort has been applied to describe the sustained post-chemotherapy damage in clinical and animal studies. However, most in vitro mechanistic studies have focused on the responses to Dox treatment while events that occur during Dox washout have not been sufficiently explored. Importantly, analysis of cardiac dysfunction performed in [47] suggested differential mechanisms of injury during Dox treatment versus the period after Dox withdrawal. In this study, we adopted the Dox treatment/washout protocol to analyze the responses of cultured endothelial cells. We report highly upregulated expression of *Tgfb2* and *Inhba* transcripts encoding the TGF-β2 and activin isoforms that activate the ALK5 and ALK4/7 receptors, respectively. Our further ChIP seq and transcriptomic analyses uncovered increased activity of Smad2/3, transcription factors in the canonical TGF-β and activin pathways. In support of this analysis, endothelial cells exhibited enhanced Smad2/3 phosphorylation and their transcriptional activation responses to TGF-β2 upon Dox washout. Thus, we identified the following factors that may be leading to the upregulated activities of the Smad2/3 pathways under these conditions, increased production of ligands activating the ALK4/5 receptors and enhanced Smad2/3 phosphorylation and transcriptional responses to a given concentration of TGF-β2. While addressing the causes of the enhanced TGF-β/Smad2/3 signaling under these experimental conditions is outside of the scope of this study most endothelial cell types express bone morphogenetic protein receptor type 2 (BMPR2) that is known to control activity of the TGF-β/Smad2/3 signaling via the Smad1/5 pathway [48]. It is plausible that the enhanced response to the TGF-β ligands in Dox treated cells is due to reduced expression or activity of BMPR2. The conceivable role of downregulation of this receptor in endothelial damage by Dox has yet to be explored.

Pharmacologic and knockout approaches used in our previous studies provided evidence that increased activity of the canonical TGF-β/Smad2/3 pathway contributes to endothelial damage and cardiac dysfunction by Dox [21, 27]. While the detrimental impact of the pathway has been demonstrated the downstream mechanisms have not been defined. TGF-β2 and activin A are critical factors inducing EndMT during cardiac valves development [49] and in cultured endothelial cells [50, 51], and, along with other members of the TGF-β family, drive EndMT and pathologic vascular remodeling in atherosclerosis, vein graft reocclusion, kidney fibrosis, and disturbed blood flow [34–36, 52]. Specifically, under conditions of disturbed blood flow in vivo, increased expression of transcripts that are typical for smooth muscle and fibroblastic lineages was detected [53]. In this study, we demonstrated that activation of mesenchymal gene expression in cultured endothelial cells by TGF-β2 was Smad3 dependent. Similarly, increased expression of mesenchymal genes was detected during Dox treatment and further upregulated upon its washout. Among them were transcripts characteristic of smooth muscle and fibroblastic lineages. Importantly, increased expression of smooth muscle and fibroblastic protein markers, transgelin and fibronectin, were detected in cardiac microvascular endothelial cells of treated mice three weeks after completion of Dox therapy. The canonical TGF-β pathway is involved in both smooth muscle [54] and myofibroblast [55] differentiation. It was not possible to resolve using the bulk RNA sequencing approach if most partially reprogrammed endothelial cells expressed the transcripts of both lineages, or there were cell subpopulations with predominant gene expression characteristic of one of these lineages. Using *Tagln*, the most highly expressed gene during our Dox washout experiments, as an example, we demonstrated that its enhanced expression is likely mediated by increased binding of Smad3 and transcriptional activation of the promoter. The causal role of the canonical TGF-β/Smad2/3 pathway was further supported in our experiments with SB, a selective ALK4/5/Smad2/3 pathway inhibitor [41]. SB has been reported to promote differentiation of human and mouse progenitor cells into endothelial lineage and maintain the differentiated status of the reprogrammed endothelial cells [56–58]. In our experiments, SB reduced transcriptional activity of the *Tagln* promoter and expression of *Tagln* and other mesenchymal markers during both Dox treatment and its washout. Thus, inhibition of the TGF-β/Smad2/3 pathway with SB effectively prevents mesenchymal reprogramming to preserve endothelial identity after completion of Dox therapy. Sustained changes in the endothelial transcriptome after Dox removal imply long-term reprogramming of the affected endothelial cells. As a DNA intercalating agent, Dox binds to nucleosome-free regions of genome containing promoters, enhancers, super-enhancers, and gene introns [59]. As a result, Dox was found to cause eviction of certain histones from DNA in several tumor cell lines that may cause the loss of their epigenetic marks [60]. Again, these studies focused on Dox treatment and not washout, and the concentrations of the drug were much higher than those in our protocols. In addition, such non-specific changes would cause general deregulation of gene expression while we report changes in expression of co-regulated and functionally related genes. Specifically, we present evidence that certain aspects of partial endothelial reprogramming by Dox are Smad2/3 dependent. The pattern of the whole genome Smad3 binding sites was altered during Dox washout, showing enrichment in fibrotic, cytoskeletal remodeling, and smooth muscle contraction pathways. These same pathways are upregulated in the endothelial transcriptome, suggesting that the described changes in Smad3 binding are functionally significant. To explain the altered pattern of gene expression observed in EndMT, epigenetic mechanisms have been implicated [61, 62]. In particular, activation of the TGF-β/Smad3 pathway promotes epigenetic modifications via DNA methylation and histone acetylation mechanisms [63, 64]. Thus, it is plausible that the Smad3 mediated pathways, including epigenetic remodeling and direct induction of certain genes (*Taglin*, for example) contribute to the sustained endothelial reprogramming after completion of Dox therapy.

Endothelial reprogramming leads to a partial loss of endothelial phenotype and compromised vascular functions. Prior exposure to Dox predisposes to atherosclerotic plaque formation in mice on a high-fat diet [65]. Likewise, studies in adult survivors of childhood cancers, most of whom received Dox treatment, revealed chronic vascular inflammation and early atherogenesis [22], processes that are predominantly driven by endothelial reprogramming [33]. In addition, clinical follow-up studies detected myocardial perfusion defects in breast cancer patients treated with Dox containing chemotherapy regimens [66, 67]. Similarly, studies on large animals have described long-lasting damage to cardiac microcirculation and large coronary arteries by Dox, leading to reduced myocardial perfusion and blunted vasodilation, respectively [25, 68]. Importantly, vascular damage preceded development of contractile dysfunction in Dox treated animals. In this study, we propose how mesenchymal reprogramming may contribute to sustained damage of cardiac vasculature. One of the mechanisms may involve significantly increased production of extracellular matrix proteins, including fibronectin and collagens, type I and type III, by the treated endothelial cells. These proteins are excreted by endothelial cells, as shown in our experiments with fibronectin, to potentially contribute to perivascular fibrosis, a hallmark of Dox cardiomyopathy [69]. Additionally, increased production of these fibrotic proteins will erode and change the composition of the endothelial basement membrane, consisting primarily of laminins 411 and 511, and collagen, type IV. Laminins, in particular, are essential for endothelial barrier function and shear stress response, and loss of laminin 511 impairs vascular permeability and vasodilation in resistance arterioles [70, 71].

Another plausible mechanism of vascular dysfunction may result from increased endothelial expression of genes characteristic of smooth muscle lineage. Alpha-tropomyosin, a protein encoded by the *Tpm1* gene, stabilizes the F-actin network by preventing its disassembly in non-muscle cells [72]. Similarly, expression of transgelin, a product of the *Tagln* gene, changes endothelial morphology from cobblestone-like to elongated and causes F-actin stabilization and cytoskeletal reorganization [73]. Our transcriptomic and ChIP seq analyses showed that, in addition to promoting these characteristic cytoskeletal changes, Dox is predicted to activate the Rho-GTPase pathways that often accompany dissolution of intercellular junctions and increased permeability of endothelial monolayers. Indeed, enhanced permeability of cardiac microvasculature in treated mice [74] and monolayers of human cardiac microvascular endothelial cells during Dox treatment in vitro [75] were previously reported. Interestingly, the described changes in endothelial cytoskeleton and barrier permeability are also induced by TGF-β treatment [76, 77]. Our work demonstrates persisted endothelial barrier dysfunction after Dox withdrawal and provides evidence that it is mediated by the canonical TGF-β pathway.

## Conclusion

As the nature of delayed cardiovascular deterioration by anticancer anthracyclines is not understood we focused in this study on the mechanisms of endothelial damage that occurs upon completion of chemotherapy. We report that enhanced activity of the canonical TGF-β/activin pathway persists after Dox removal and contributes to sustained partial endothelial-to-mesenchymal reprogramming, cytoskeletal derangement, and compromised endothelial function. Suppression of the pathway alleviates mesenchymal reprogramming and functional deterioration of treated endothelial cells. Vascular damage and inappropriate activation of the TGF-β/activin pathway accompany multiple cancer treatments besides anthracyclines [19, 28, 29, 78, 79]. In addition, this pathway significantly contributes to cardiovascular remodeling and dysfunction in patients and animal models of cardiac hypertrophy, myocardial infarction, and heart failure [80–83]. Thus, the mechanistic insights from our studies with Dox will likely apply, at least partially, to a broad range of cardiovascular conditions. While numerous studies have focused on the effects of the TGF-β pathway on fibroblasts, immune cells, and cardiomyocytes, this work draws attention to the role of the pathway in sustained endothelial reprogramming and dysfunction. While utilizing inhibitors of the canonical TGF-β pathway appears effective against certain aspects of endothelial damage the approach will have to be further tested using other cardiovascular cell types and models of Dox cardiomyopathy in order to overcome the limitation of this study that was performed using preclinical models [84]. Further efforts are warranted to address the cell specific and context dependent actions of the highly multifunctional TGF-β superfamily in order to define its role in chemotherapy-induced cardiomyopathies and other cardiovascular diseases.

## Supplementary Information

Supplementary Material 1

## Data Availability

The datasets supporting the conclusions of this article are included within the article and its Supplementary Material file. RNA-seq and ChIP-seq data were deposited into the Gene Expression Omnibus database under accession numbers GSE300789 and GSE300800.
