# Supplementary material for: Sustained mesenchymal reprogramming of endothelial cells after completion of chemotherapy
Source: Cardiooncology. 2025 Dec 29;11:116. doi: 10.1186/s40959-025-00413-7 (PMC12751855; doi:10.1186/s40959-025-00413-7)
Supplement: Supplementary file 1 — Supplementary Material 1 [file 40959_2025_413_MOESM1_ESM.pdf]

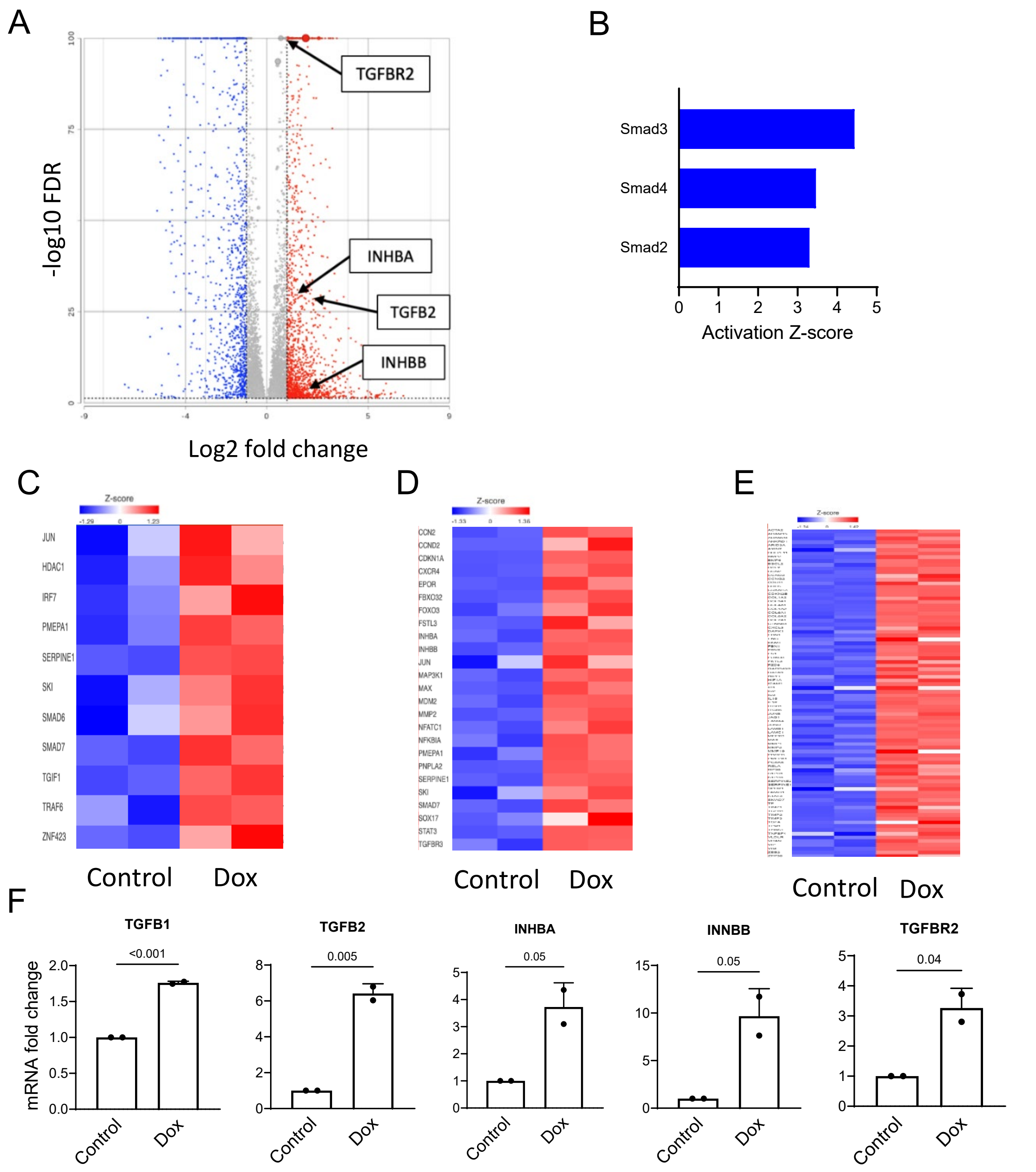

Suppl. Fig. 1. Transcriptomic analysis of the TGF-β/activin/Smad2/3 pathways in endothelial cells during Dox treatment. Total RNA from control and treated with Dox (16 nM) for 48 hours HUVEC samples was used for RNA-seq analysis. (**A**) A volcano plot presenting significantly different differentially expressed protein coding genes in Dox versus control samples. Positions of the TGF-β/activin related transcripts are indicated with arrows. (**B**) Significantly different differentially expressed protein coding gene lists were analyzed with IPA to identify the upstream regulators related to the TGF-β/activin pathways and their predicted degrees of activation using Z-scores. (**C**), (**D**), and (**E**) Heatmaps presenting Z-scores for the TGF-β and activin pathways related transcripts, and Smad3 target genes, respectively. (**F**) Validation of the transcriptomic results using qPCR analysis of expression of the selected TGF-β/activin pathways related transcripts. *Tgfb1*, transforming growth factor beta 1; *Tgfb2*, transforming growth factor beta 2; *Inhba*, inhibin subunit beta A; *Inhbb*, inhibin subunit beta B; *Tgfbr2*, transforming growth factor beta receptor 2. The *p* values for the unpaired two-tailed *t*-test are shown.

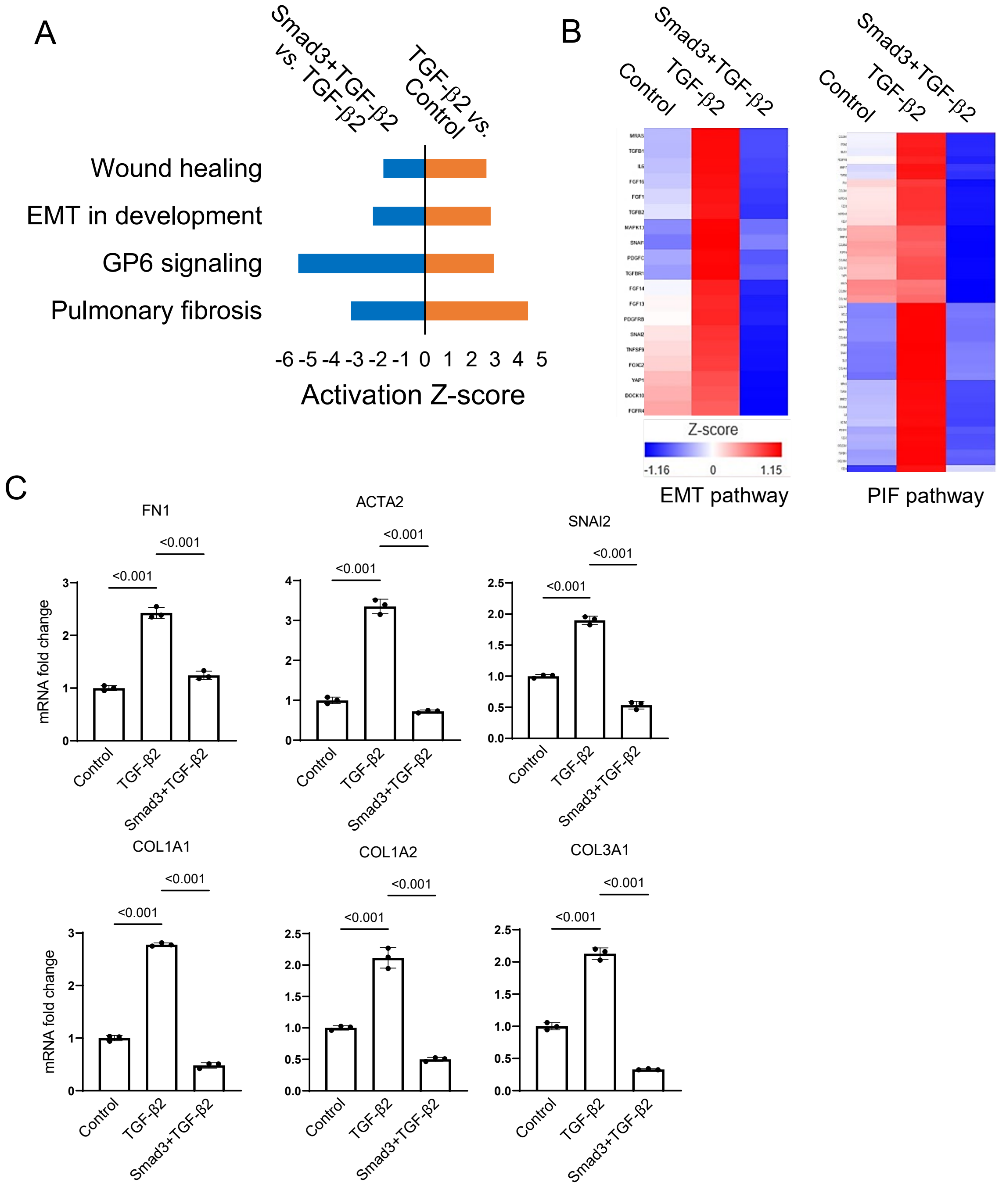

Suppl. Fig. 2. Transcriptional analysis of endothelial responses to TGF-β2. Human cardiac microvascular endothelial cells were transduced with lentiviral particles expressing Smad3 or scrambled (control) short hairpin RNAs, as described in (19). Cell lines were characterized therein. Cells were starved in growth factor free endothelial basal media for 4 hours and treated with 0.3 ng/ml TGF-β2 for 16 hours. Total RNA was isolated for sequencing and transcriptomic analysis. (A) Statistically significant differentially expressed genes were uploaded to IPA to characterize enriched canonical pathways in TGF-β2 treated versus untreated samples. Activation Z-scores are shown for the scrambled (control) TGF-β2 versus untreated control; and Smad3 knockdown TGF-β2 versus control TGF-β2 pairwise comparisons. (B) Heatmaps presenting Z-scores for the epithelial-to-mesenchymal (EMT) and pulmonary idiopathic fibrosis (PIF) pathways related transcripts. (C) Validation of the transcriptomic results using qPCR analysis of expression of selected mesenchymal/fibrotic genes. *Fn1*, fibronectin 1; *Acta2*, actin alpha 2, smooth muscle; *Snai2*, snail family transcriptional repressor 2; *Col1a1*, collagen type I alpha 1 chain; *Col1a2*, collagen type I alpha 2 chain; *Col3a1*, collagen type III alpha 1 chain. The *p* values for the one-way ANOVA followed by Sidak correction method are shown.

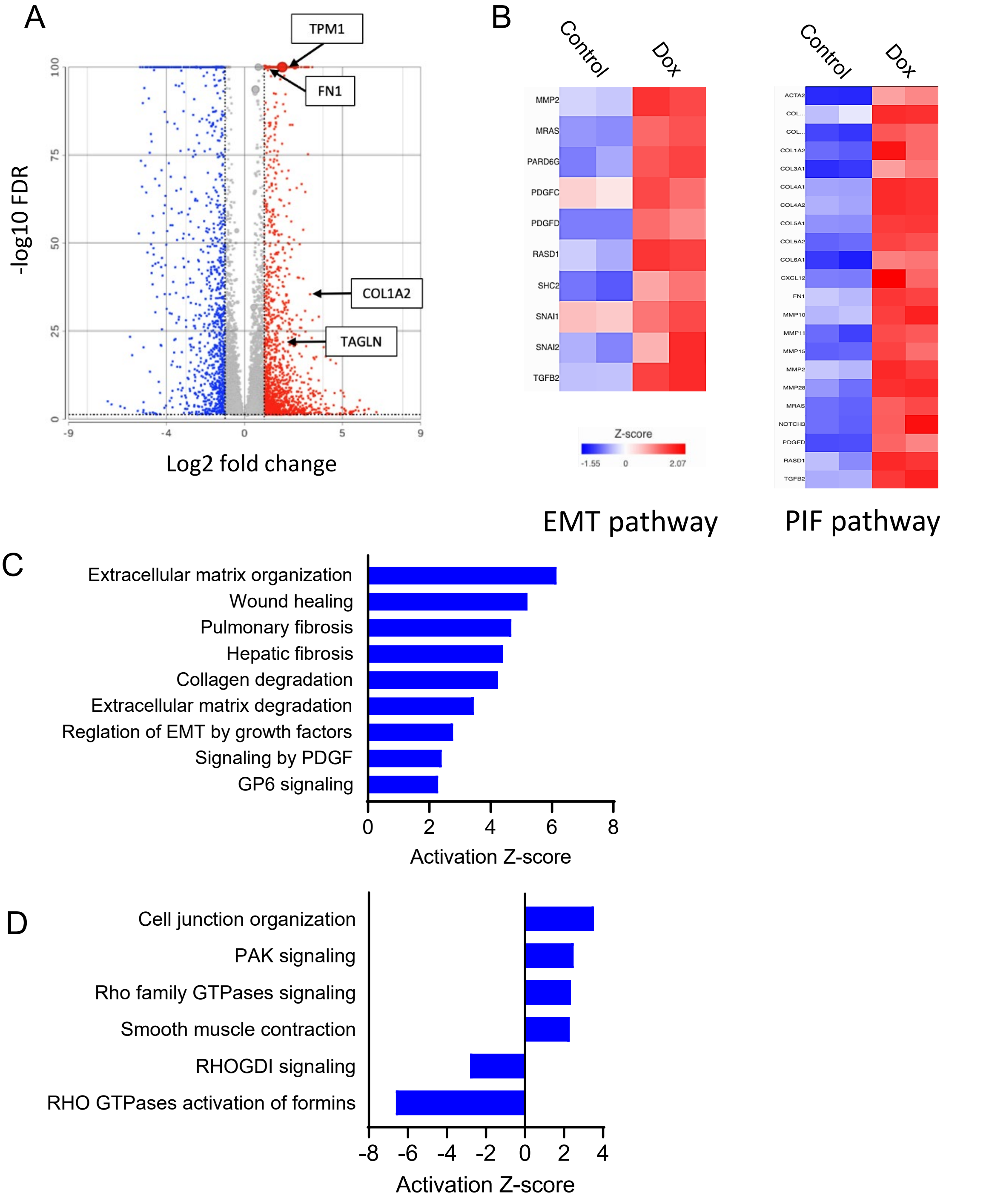

Suppl. Fig. 3. Transcriptional analysis of mesenchymal activation in endothelial cells during Dox treatment. Total RNA from control and treated with Dox (16 nM) for 48 hours HUVEC samples was used for RNA-seq analysis. (**A**) A volcano plot presenting significantly different differentially expressed protein coding genes in Dox versus control samples. Positions of the mesenchymal transcripts are indicated with arrows. (**B**) Heatmaps presenting Z-scores for the epithelial-to-mesenchymal (EMT) and pulmonary idiopathic fibrosis (PIF) pathways related transcripts. (**C**) and (**D**) Significantly different differentially expressed protein coding gene lists were analyzed with IPA to identify the upstream regulators related to the fibroblastic/EMT and smooth muscle/cytoskeletal regulation pathways, respectively, and their predicted degrees of activation using Z-scores.

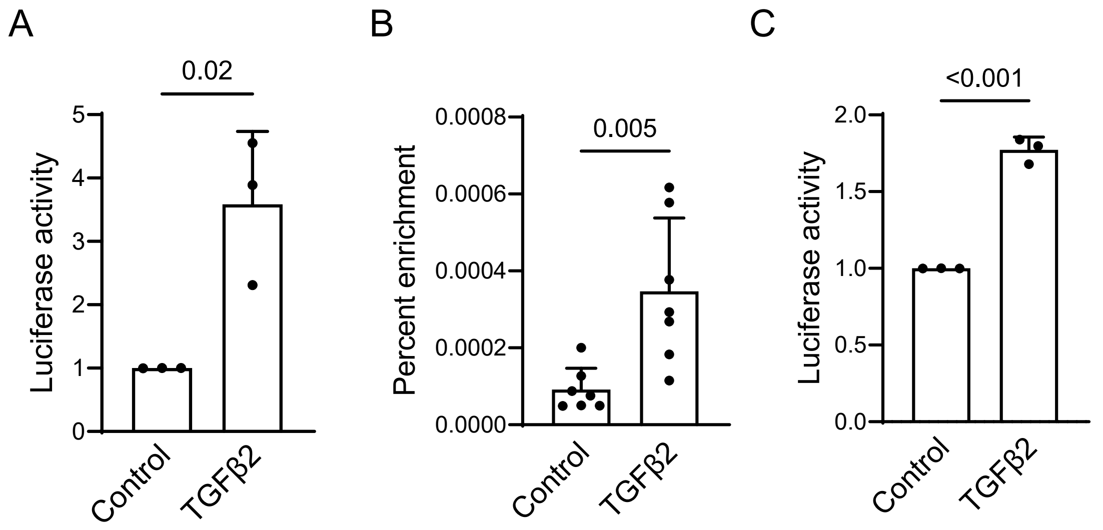

Suppl. Fig. 4. TGF-β2 enhances Smad3 transcriptional activity and its binding to the *Tagln* promoter. The results of the positive control experiments with TGF-β2 are shown. HUVEC cultures were starved for 4 hours in the growth factor free endothelial basal media and treated with TGF-β2. (**A**) Cells were transfected with $CAGA_{12}$-luciferase and Renilla luciferase plasmids, treated with 1 ng/ml TGF-β2 for 24 hours, and lysed for Firefly and Renilla luciferase activity measurements. Results are presented as Firefly/Renilla luciferase activity ratios and normalized to control. n=3 independent experiments. (**B**) Cells were starved, treated with 0.3 ng/ml TGF-β2 for 1 hour, and processed according to the ChIP-PCR protocol. N=7 independent experiments. (**C**) Cells were transfected with *Tagln* promoter-luciferase and Renilla luciferase plasmids, treated with 1 ng/ml TGF-β2 for 24 hours, and lysed for Firefly and Renilla luciferase activity measurements. Results are presented as Firefly/Renilla luciferase activities ratios normalized to control. n=3 independent experiments. The *p* values for the unpaired two-tailed *t*-test are shown.

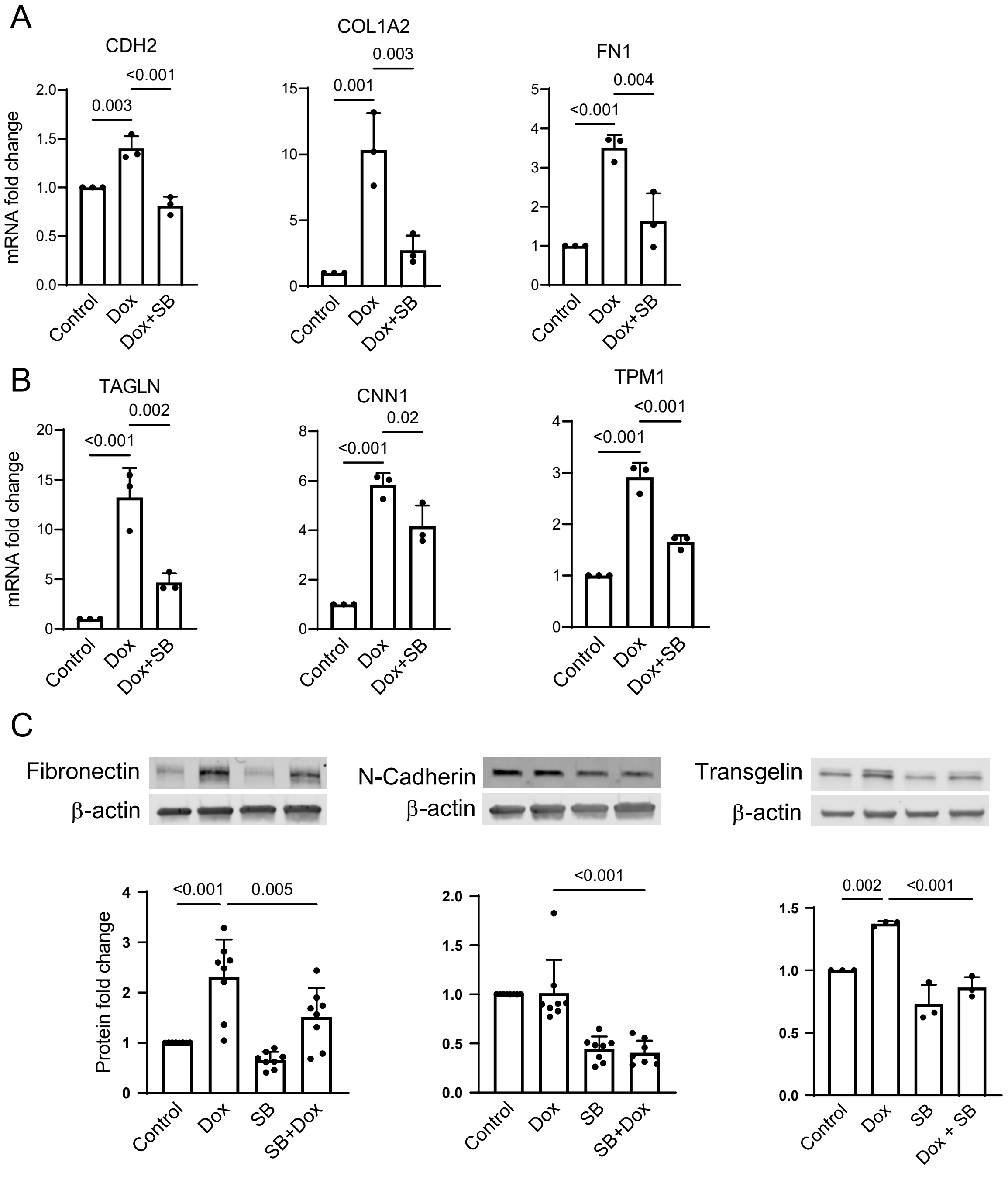

Suppl. Fig. 5. ALK4/5/7 inhibitor suppresses mesenchymal activation in endothelial cells during Dox treatment. HUVEC were treated with Dox (16 nM) and/or SB (1 μM) for 48 hours and lysed for total RNA isolation (**A** and **B**) or protein extraction (**C**). (**A** and **B**) Expression of mesenchymal transcripts characteristic of fibroblastic and smooth muscle lineage, respectively. n=3 independent experiments. (**C**) Expression of mesenchymal protein markers fibronectin, N-cadherin, and transgelin (n=3 to 8 independent experiments). The *p* values for the one-way ANOVA followed by Sidak correction method are shown.

Suppl. Table 1. Effect size values for expression of mesenchymal transcripts in endothelial cells upon Dox washout: Effects of concurrent SB versus SB during washout

**Transcript**	Cdh2	Col1a2	Fn1	Tagln	Cnn1	Tpm1
SB concurrent only	1.10	0.74	1.51	2.26	4.62	1.11
SB during WO only	2.73	2.16	1.74	2.42	3.5	5.76

Effect size was calculated for datasets presented in **Fig. 5F** (concurrent SB only followed by no treatment Dox washout) and **Fig. 5G** (SB present during Dox washout [WO]) and presented as Cohen's *d* parameter values.
